# The immediate and short-term effects of dynamic taping on pain, endurance, disability, mobility and kinesiophobia in individuals with chronic non-specific low back pain: A randomized controlled trial

**DOI:** 10.1371/journal.pone.0239505

**Published:** 2020-09-29

**Authors:** Khalid A. Alahmari, Kanagaraj Rengaramanujam, Ravi Shankar Reddy, Paul Silvian Samuel, Jaya Shanker Tedla, Venkata Nagaraj Kakaraparthi, Irshad Ahmad

**Affiliations:** Department of Medical Rehabilitation Sciences, College of Applied Medical Sciences, King Khalid University, Abha, Kingdom of Saudi Arabia; Universidade Federal do Rio Grande do Sul, BRAZIL

## Abstract

Evidence suggests that the application of Kinesio Tape (KT) on patients with chronic non-specific low back pain (CNLBP) is inconclusive. Dynamic tape (DT) is a relatively new treatment technique, which is increasingly being used as an adjunctive method to treat musculoskeletal problems. To our knowledge, no study has investigated the application of DT in individuals with CNLBP. To compare the immediate and short-term effects of DT versus KT and no tape among patients with CNLBP on pain, endurance, disability, mobility, and kinesiophobia. Forty-five patients with CNLBP were randomly assigned to 1 of 3 groups. Outcomes were measured at baseline, immediately, and on the third day post-application of tapes. The primary outcomes of pain, endurance, and disability were measured through the visual analog scale (VAS), Biering–Sorensen test, and Oswestry disability index (ODI), respectively. Secondary outcome measures of mobility and kinesiophobia were measured using the modified–modified Schober test and the Tampa Scale of Kinesiophobia, respectively. No significant immediate and short-term differences were found between DT and KT in pain, disability, mobility, and kinesiophobia. Improved back extensor endurance was observed for the DT group than KT (*p* = 0.023) and control group (*p* = 0.006). The application of DT may result in improvements only in back extensor endurance among individuals with CNLBP. This finding suggests that DT controls the processes that lead to back muscle fatigue.

## Introduction

Low back pain (LBP) is a critical public health condition that is associated with a high rate of absenteeism from work, disability, and the frequent use of health services [1]. The most common type of LBP is non-specific LBP, which affects people of all ages and is an important contributor to disease burden worldwide [2]. Chronic non-specific low back pain (CNLBP) manifests as pain, stiffness, muscular tension, restriction on mobility and disability between the costal margin and inferior gluteal folds lasting for 12 weeks or more [3], and is considered one of the leading causes of disability across an entire life-span [4].

The current literature supports several options for the treatment of LBP that vary according to the duration of symptoms [5] and the classification of this condition [6]. These treatment options include educational programs [7], behavioral therapy [8], cognitive therapy [9], medication [10], electrophysical agents [11], manual therapy [12], kinesio taping [13], general exercises [14], and specific spinal stabilization exercises [15]. Although the treatments above have been widely used, at best, they demonstrate a limited effect, with recurrences typically noted [16]. Therefore, more effective therapeutic interventions are critically required to manage the LBP.

Taping is one of the therapeutic interventions practiced by physical therapists and other members in the rehabilitation team. Sports injuries, different type of clinical conditions and disorders in the spine are prevented or managed by taping techniques [17, 18]. Numerous types of tape and their associated application methods are available, with different underlying philosophies regarding their modes of action. Kinesio tape (KT), Dynamic tape (DT), rigid tape, micropore tape, athletic tape, and many other types are available to manage and/or rehabilitate injuries.

DT is a relatively new treatment technique that is increasingly becoming an adjunctive method to treat musculoskeletal problems. In 2009, musculoskeletal physiotherapist Ryan Kendrick created DT, which is made up of a visco-elastic nylon and lycra blend material and can stretch in four directions, has strong elastic resistance and recoil, as well as a high degree of stretch (more than 200%) with no rigid endpoint and visco-elastic properties. The primary mode of action of DT is mechanical (deceleration of eccentric work, load absorption, and assistance of movement), while the second mode of operation is neurophysiological [19].

KT was developed in 1973 by the Kenzo Kase and is applied in an elastic taping method to the patients’ skin under tension. It can be longitudinally extended up to 140% of its original length, producing a lesser mechanical restraint and less mobility restriction than conventional tape. KT has been reported to correct joint malalignment, provide support for muscles, activate the endogenous analgesic system, and eliminate congestion fluids [20].

Studies have extensively analyzed the effectiveness of KT application in CNLBP, with mixed results [21–25]. A systematic review [25] concluded that KT is not a substitute for routine physical therapy or exercise. We need to examine whether another tape, such as DT, has the same effect. Studies with longer-term follow-up are warranted to evaluate the effect of DT. However, since DT’s feasibility is currently unknown on CNLBP, it is valuable to assess the immediate and short-term effects in a study before conducting a larger randomized controlled trial with a longer-term follow-up. Studies have already analyzed the immediate and short-term application of KT on pain [26–29], disability [26–29], endurance [27], and range of motion (ROM) [26, 30, 31] among subjects with CLBP and produced with mixed results. Among the available limited studies of DT application on musculoskeletal problems, the DT produced promising results on outcomes. Studies have concluded with the application of DT decrease pain and improve the mechanical effect on greater trochanteric pain syndrome (GTPS) [32]; decreases pain, improve disability and ROM on the post-operative shoulder [33]; decreases pain and improve disability on chronic mechanical neck pain [34]; and decrease load on lower limb muscles as well as improve movement pattern and quality on subjects with patellofemoral pain [35].

Thus, we hypothesized that the immediate and short-term effects of DT are better than KT and no tape in terms of the reduction of pain and the improvement of endurance, disability, mobility, and kinesiophobia. However, no research has evaluated the impact of DT on LBP, and thus, the present study aims to determine the effect of DT on the treatment of CNLBP.

## Materials and methods

### Design and setting

A randomized controlled trial was conducted at the out-patient physical therapy clinic, King Khalid University located in Saudi Arabia.

### Participants

Over 2-months, male patients referred from an orthopedic surgeon to physical therapy in the out-patient physical therapy department were screened for eligibility. Inclusion criteria were as follows: patients between 18 to 60 years of age, referred to physical therapy with a diagnosis of LBP, with a minimum duration of symptoms of more than 12 weeks, minimum pain intensity score of 3 and above on a 10 cm long visual analog scale, and a disability rating of at least 20% on the Oswestry disability index (ODI) [36] at the time of assessment. Exclusion criteria were as follows: any contraindication for taping, pain radiating to the knee, a known or suspected serious congenital or acquired spinal pathology, spinal surgery, lumbar disc herniation, spinal deformity, rheumatoid arthritis or spondyloarthropathy, and an inability to tolerate the Biering–Sorensen test [37]. Ethical approval was obtained to the study protocol from the Institutional Review Board at King Khalid University, Saudi Arabia (ECM#2019–71). Written informed consent was obtained from all participants prior to study enrollment, and all rights were protected. This trial was registered at the ISRCTN Registry, www.isrctn.com (ISRCTN12043220).

### Allocation

**Fig 1:**
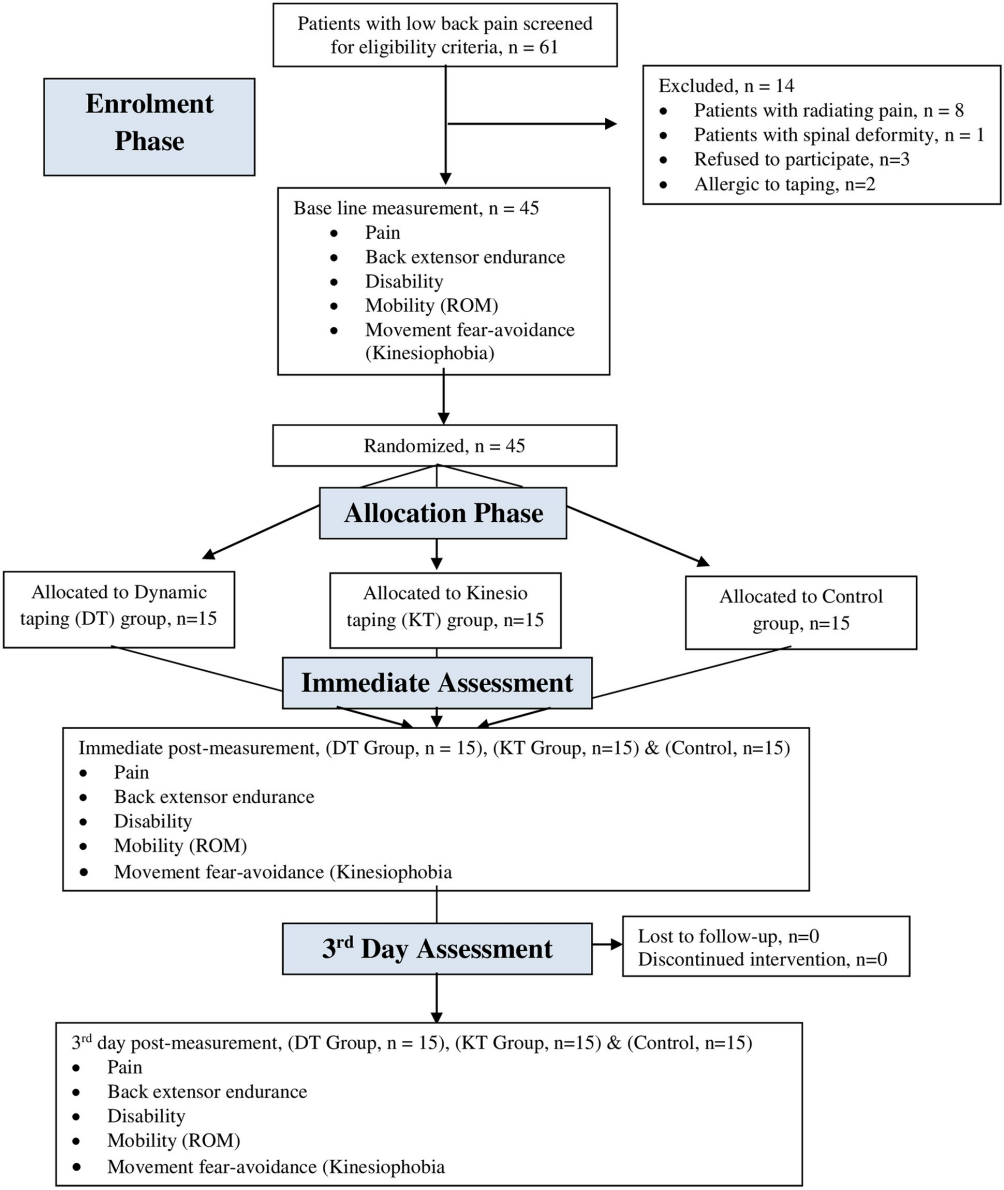
Study flow diagram. ROM = range of motion.

Following the baseline examination, by using the method on the website http://randomizer.org/ (Social Psychology Network, Connecticut, USA), participants were randomly assigned into the DT group (n = 15), KT group (n = 15), and control group (n = 15). Concealed allocation was performed using a computer-generated block randomized table of numbers (1 for DT group, 2 for KT group, and 3 for control group) created before the start of data collection by a researcher (Researcher 1) who was not involved in the recruitment or treatment of patients. After that, the random numerical sequence was placed in sealed opaque envelopes. Another researcher (Researcher 2), blinded to the baseline examination, opened an envelope and proceeded with treatment according to the group assignment. An independent assessor who not known the study’s hypothesis and methods was blinded to the treatment group assessed the outcome measures before the tape application, 15 minutes after tape application and on the third day of post-tape application. A flow chart of the participants during the selection, follow-up, and analysis phases is provided in Fig 1. The authors changed from the study protocol the duration between tape application and immediate post-application of measurement.

In the study protocol, we had mentioned earlier that the immediate post-measurement would be done 2 hours after the tape application. The authors changed the study protocol to only 15 minutes duration between the tape application and immediate post-application outcome measurement because patients were unwilling to wait for 2 hours post taping. A 15 minutes rest interval was sufficient to measure the outcomes among subjects with LBP after tape application, according to a study done by Velasco-Roldan et al. [31].

Due to the presence of a group without tape, it was not possible for the participants to be blinded to the treatment. However, before any analysis was performed, the data were coded by another researcher (Researcher 4), as well as statistical analysis being performed by one more researcher (Researcher 5), who was also blinded.

### Outcome measures

Baseline evaluation included demographic data, medical history, and physical examination. Demographic data included patients’ age, body weight (kg), height (cm), and body mass index (weight in kilograms divided by height in meters squared), marital status, education, employment status, smoking, duration and recent episode of the condition, as well as regular exercise. The routine physical examination performed to determine the subject did not include LBP with a neurological origin.

The outcomes were assessed prior to tape application, 15 minutes after tape application with tape in situ, and on the third day of post tape application without tape. The primary outcome measures were pain, back extensor endurance, and disability. Pain was assessed with self-report measures of visual analog scale (a 10 cm scale, where 0 represented no pain and ten represented unbearable pain), which is a reliable and valid instrument to measure pain among patients with chronic LBP [38].

Back extensor endurance was measured in seconds by using the Biering–Sorensen test, which is considered to have sufficient test–retest reliability (intraclass correlation coefficient (ICC) = 0.88) [37] for assessing isometric back extensor muscle endurance [39]. Participants were positioned on a treatment table in prone with the lower half of their body secured with three straps. For testing, the participant’s ability to maintain a horizontal position was timed using a stopwatch/timer, and standardized verbal encouragement was provided at 30-second intervals. The participant was placed in the starting position for the test, prone on a plinth with the upper edge of the iliac crests aligned with the edge of the table. A second hydraulic table was transversely placed at the same height to the first one under the trunk and upper body, so that the participants were supported completely in a prone position prior to the initiation of the test. The lower limbs were fixed to the table in full extension, together, and with ankles in plantar flexion using three straps perpendicular to the midline. The first strap was located at the level of the greater trochanter, the second at the level of the popliteal fossa, and the last at the level of the Achilles tendon insertion, as close as possible to the malleoli. A digital inclinometer was fixed to the participant’s inter-scapular region by an elastic strap around the chest and was used to measure changes in flexion or extension of the subject during the test.

At the initiation of the test, the second table was lowered, and the subject was asked to place their arms across their chest and maintain a neutral spinal position. The timer was started as soon as their arms were positioned across their chest, and the participant maintained this position without assistance. However, at no point was either the researcher or participant aware of the amount of time that had passed, as this is a factor that directly influences test results. During the test, between 5^0^ of extension and 5^0^ of flexion in the inclinometer was permitted. The test ended when any part of the participant’s upper limb touched the table or when they were unable to recover the test position even with verbal encouragement. At this point, the timer was stopped and the test was finished [37].

Participants’ disability was assessed using the English [40] or Arabic version [36] of the ODI. This is a self-rating questionnaire used to evaluate functional physical disability. It includes ten sections of six propositions, each rated on a 0–5 scale, while relative values are reported (total score / total possible score X 100%). A higher score indicates a worse disability [40]. Scoring methods followed as per the instructions given in the questionnaire. The English version of test–retest reliability (*r* = 0.83–0.99) and interclass correlation coefficient (0.84–0.94) are high [41], while the Arabic version of ODI has excellent intra-rater reliability (ICC = 0.99) [36].

The secondary outcome measures were mobility and movement fear-avoidance (kinesiophobia). The mobility of the spine (spinal flexion ROM) was measured using the modified–modified Schober’s test (MMST). The measurement was done with the participant standing erect, knees extended, arms relaxed at the sides, and body weight centered. The inch tape and marker were used to make three markings on the skin overlapping the lumbosacral spine. The first mark (middle line) was made at the lumbosacral junction, as specified by the midpoint between the two posterior superior iliac spines (PSIS) at the S2 level. The second point mark (superior line) was made 10 cm above the lumbosacral junction. Finally, the third point mark (inferior line) was made 5 cm below the lumbosacral junction. The participant was instructed to bend forward as much as possible, and the additional distance between the second and third marks was measured. The original length (15 cm) was subtracted from the final length of trunk flexion to obtain the extent of trunk flexion [42]. The MMST showed moderate validity (*r* = 0.67) and excellent reliability (intra-rater, ICC = 0.95, and inter-rater, ICC = 0.91) [42] to measure spinal flexion ROM.

The movement fear-avoidance (kinesiophobia) was assessed using the Tampa scale of kinesiophobia (TSK). This is a self-reported questionnaire consisting of 17 items, using a 4-point Likert scale, which was developed to measure the fear of movement or (re)injury among patients with LBP. The total score ranges between 17 and 68, while a high score on TSK indicates a high degree of kinesiophobia [43]. Both the English and Arabic version of TSK were used in this study. The English version of TSK has good test-retest reliability (r = 0.29–0.69) and internal consistency (α = 0.84) [43], while the Arabic version of TSK has high intra-observer reliability (ICC = 0.86) and internal consistency (α = 0.0.87) [44] for CLBP.

### Intervention

**Fig 2:**
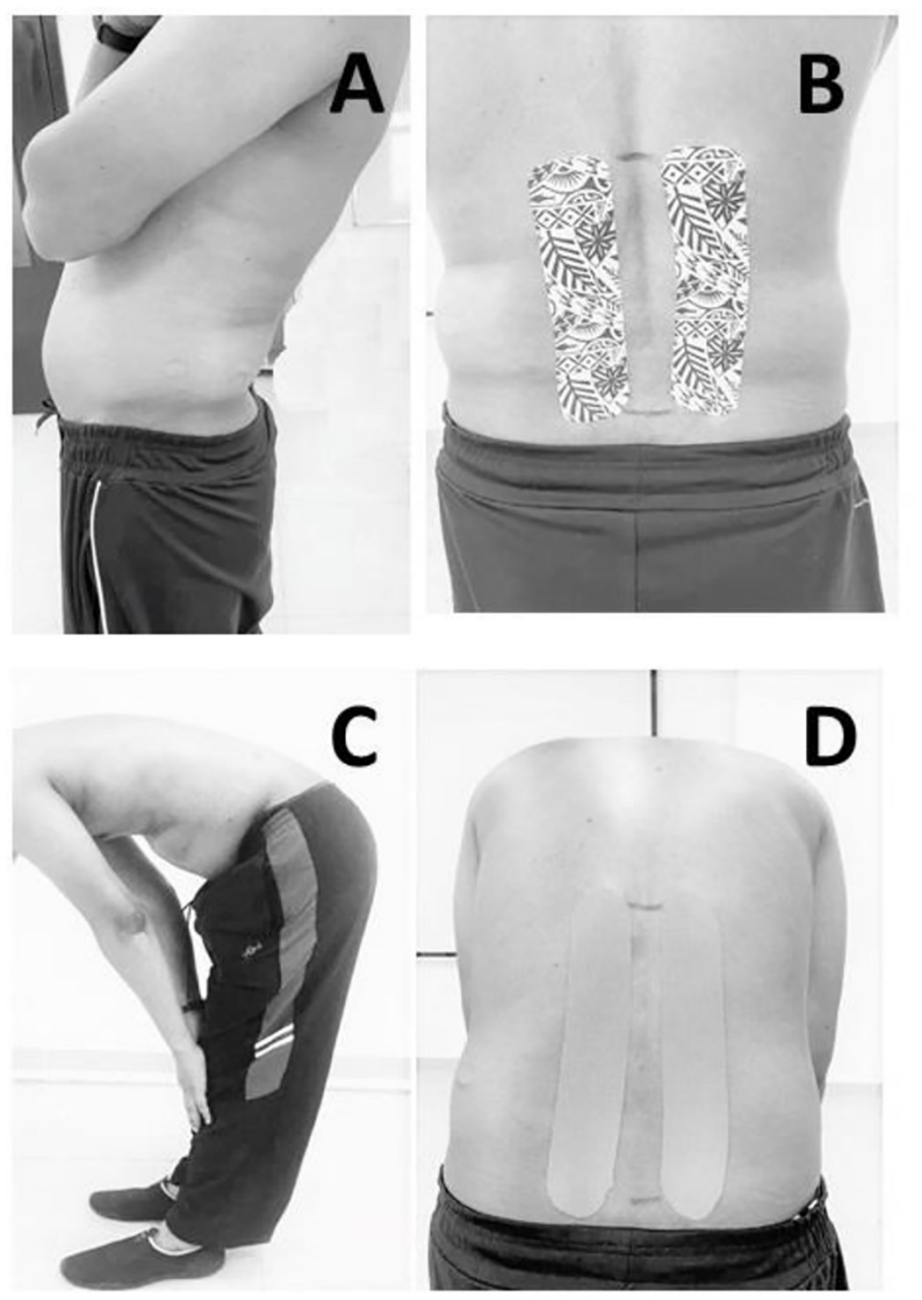
I-shaped dynamic tape (A. Taping position, B. Application of tape) and Kinesio tape (C. Taping position, D. Application of tape).

After completing the screening procedures to include the study participants, a piece of DT & KT was applied on the right and left side forearm, respectively. The absence of an allergic reaction was confirmed after 24 hours of tape application, and the participants were excluded from the study if any allergic reaction was present. After completing the baseline measurement, the participants were randomized into three groups. Participants were taped according to their group allocation by the same researcher. Once the participant was standing in a comfortable position, the PSIS and T12 (thoracic) vertebrae were carefully marked on the body’s surface. The participants were asked to extend the back as far as possible; during this time, two DT “I” strips were applied parallel to the spine without any stretch, both right and left, from the PSIS to T12 vertebrae (Fig 2A and 2B). Participants in the KT group were asked to flex the spine as much as possible. While doing so, two KT “I” strips were applied parallel to the spine with an approximately 10–15% slight stretch in both the right and left side, from the PSIS to T12 vertebrae [26] (Fig 2C and 2D). The individual in this manuscript has given written informed consent to publish these case details. However, the control group did not receive any tape. After completion of the trial, all participants underwent routine physical therapy care.

### Sample size calculation

*A priori* power analysis was performed using GPower 3.1.9.4 software. Prior randomized trials [21, 45, 46] have estimated effect sizes (0.22–0.64) for changes in back pain using VAS with KT application in patients with chronic LBP. To generate a current sample size estimate, we used an effect size of 0.22 with Cohen’s d, an alpha of 0.05, and power of 80%. A sample size of 15 participants per treatment group was used to detect a group-by-time interaction.

### Statistical analysis

Data were analyzed with SPSS Version 21.0 (IBM-SPSS Inc., Armonk, NY). The normality of distribution of all variables was verified using the Shapiro–Wilk test. The demographic and baseline characteristics between groups were compared by the one-way analysis of variance (ANOVA) for parametric variables and the chi-square test for nonparametric variables. A mixed-methods ANOVA (3 x 3) was used to analyze the main effect (group effect and time effect) and time x group interaction. All statistics were conducted using pre-protocol and intention-to-treat analyses. The effect size was calculated using η_p_^2^, which reports the proportion of the total difference within the dependent variables. When the effect of the test was significant, a Tukey post hoc test was used to identify the difference, while the *p*-value <0.05 was considered significant.

## Results

A total of 61 subjects with CNLBP were selected from December 15^th^, 2019 to February 15^th^, 2020. The final sample included 45 subjects with a mean age of 37.2 ± 8.97 (range 20–53 years). No losses to follow-up were recorded during the data collection and analysis phase. In the demographic and baseline comparison between groups, no differences were found among the participants (*p*> 0.05) (Table 1).

**Table 1:** Demographic and baseline characteristics of the groups*.

Variable	DT Group (n = 15)	KT Group (n = 15)	Control Group (n = 15)	*p* value
Age, y	37.47 ± 9.09	36.60 ± 9.06	37.53 ± 9.36	0.953
Weight, kg	71.73 ± 7.36	72.00 ± 7.37	71.87 ± 6.73	0.995
Height, m	1.69 ± 0.06	1.71 ± 0.06	1.69 ± 0.06	0.702
BMI, kg/m^2^	25.14 ± 2.47	24.79 ± 2.74	25.22 ± 2.71	0.891
Marital status, n (%)				1.00
Single	2 (13.3)	3 (20)	2 (13.3)	
Married	13 (86.7)	12 (80)	13 (86.7)	
Education, n (%)				0.938
Undergraduate	1 (6.7)	2 (13.3)	1 (6.7)	
Graduate	6 (40)	8 (53.3)	7 (46.7)	
Master degree	6 (40)	3 (20)	5 (33.3)	
PhD	2 (13.3)	2 (13.3)	2 (13.3)	
Employment status, n (%)				0.407
Sedentary work	14 (93.3)	14 (93.3)	12 (80)	
Heavy work	1 (6.7)	1 (6.7)	3 (20)	
Smoking, n (%)	4 (26.7)	2 (13.3)	5 (33.3)	0.431
Duration of LBP, mo	12.33 ± 5.51	14.80 ± 6.03	14 ± 6.40	0.521
Recent episodes of LBP, n (%)	13 (86.7)	14 (93.3)	14 (93.3)	0.760
Use of medication, n (%)	2 (13.3)	3 (20)	1 (6.7)	0.562
Regular exercise, n (%)	4 (26.7)	4 (26.7)	4 (26.7)	1.00
Pain (VAS 0–10 cm)	5.8 ± 0.87	5.71 ± 0.88	5.59 ± 0.72	0.791
Endurance (sec)	50.99 ± 11.07	49.06 ± 10.34	52.69 ± 8.32	0.611
Disability (0–100%)	28.13 ± 4.24	29.60 ± 5.19	28.93 ± 4.19	0.681
Mobility (cm)	3.36 ± 0.45	3.38 ± 0.45	3.39 ± 0.44	0.979
Kinesiophobia (17–68)	38.40 ± 2.06	39.80 ± 3.61	37.93 ± 1.75	0.137

DT = dynamic tape, KT = kinesio tape, VAS = visual analog scale.

*Values are mean ± SD unless otherwise indicated.

Table 2 shows the mean difference and effect size between groups (95% CI) in the three different assessments (pre, immediately post, and third day post) for all the outcome variables. There was significant improvement between the DT and KT groups compared with the CG.

**Table 2:** Mean differences between groups [95% confidence interval (CI)] and *p*-value at pre, immediately post and 3^rd^ day post for the variables.

Variables	Groups	Pre	Immediately post	Effect size (Immediate post)	3^rd^ Day post	Effect size (3^rd^ Day post)
Pain (VAS 0 to 10)	DT x KT	0.09 (-0.66, 0.85)	-0.81 (-1.57, -0.06)	0.187	-0.87 (-1.66, -0.08)	0.189
		1.000	0.031*		0.027*	
	DT x C	0.21 (-0.55, 0.96)	-1.91 (-2.67, -1.16)	0.621	-2.41 (-3.19, -1.62)	0.689
		1.000	<0.001*		<0.001*	
	KT x C	0.11 (-0.64, 0.87)	-1.10 (-1.86, -0.35)	0.318	-1.54 (-2.33, -0.75)	0.478
		1.000	0.002*		<0.001*	
Endurance (sec)	DT x KT	1.93 (-7.17, 11.01)	9.99 (1.59, 18.39)	0.223	17.15 (7.99, 26.31)	0.412
		1.000	0.015*		<0.001*	
	DT x C	-1.71 (-10.79, 7.38)	13.44 (5.04, 21.83)	0.356	22.53 (13.37, 31.69)	0.562
		1.000	0.001*		<0.001*	
	KT x C	-3.63 (-12.72, 5.45)	3.45 (-4.95, 11.84)	0.041	5.38 (-3.78, 14.54)	0.083
		0.973	0.935		0.451	
Disability (0–100%)	DT x KT	-1.47 (-5.63, 2.69)	-1.20 (-5.53, 3.13)	0.015	-4.80 (-8.62, -0.98)	0.412
		1.000	1.000		0.010*	
	DT x C	-0.80 (-4.96, 3.36)	-1.33 (-5.66, 2.99)	0.025	-7.60 (-11.42, -3.78)	0.562
		1.000	1.000		<0.001*	
	KT x C	0.67 (-3.49, 4.83)	-0.13 (-4.46, 4.19)	0.000	-2.80 (-6.62, 1.02)	0.083
		1.000	1.000		0.225	
Mobility (cm)	DT x KT	-0.02 (-0.42, 0.38)	-0.01 (-0.43, 0.40)	0.000	1.17 (0.66, 1.68)	0.493
		1.000	1.000		<0.001*	
	DT x C	-0.03 (-0.44, 0.37)	0.09 (-0.32, 0.51)	0.011	1.56 (1.05, 2.07)	0.728
		1.000	1.000		<0.001*	
	KT x C	-0.01 (-0.42, 0.39)	0.11 (-0.31, 0.52)	0.015	0.39 (-0.12, 0.89)	0.112
		1.000	1.000		0.196	
Kinesiophobia (17–68)	DT x KT	-1.40 (-3.77, 0.97)	-2.20 (-4.55, 0.15)	0.137	-3.67 (-5.99, -1.34)	0.289
		0.445	0.074		0.001*	
	DT x C	0.47 (-1.91, 2.84)	-1.33 (-3.69, 1.02)	0.112	-4.20 (-6.53, -1.87)	0.523
		1.000	0.496		<0.001*	
	KT x C	1.87 (-0.51, 4.24)	0.87 (-1.49, 3.22)	0.024	-0.53 (-2.86, 1.79)	0.012
		0.169	1.000		1.000	

VAS = visual analog scale, DT = dynamic tape, KT = kinesio tape, C = control.

* Significant difference: p <0.05.

**Fig 3:**
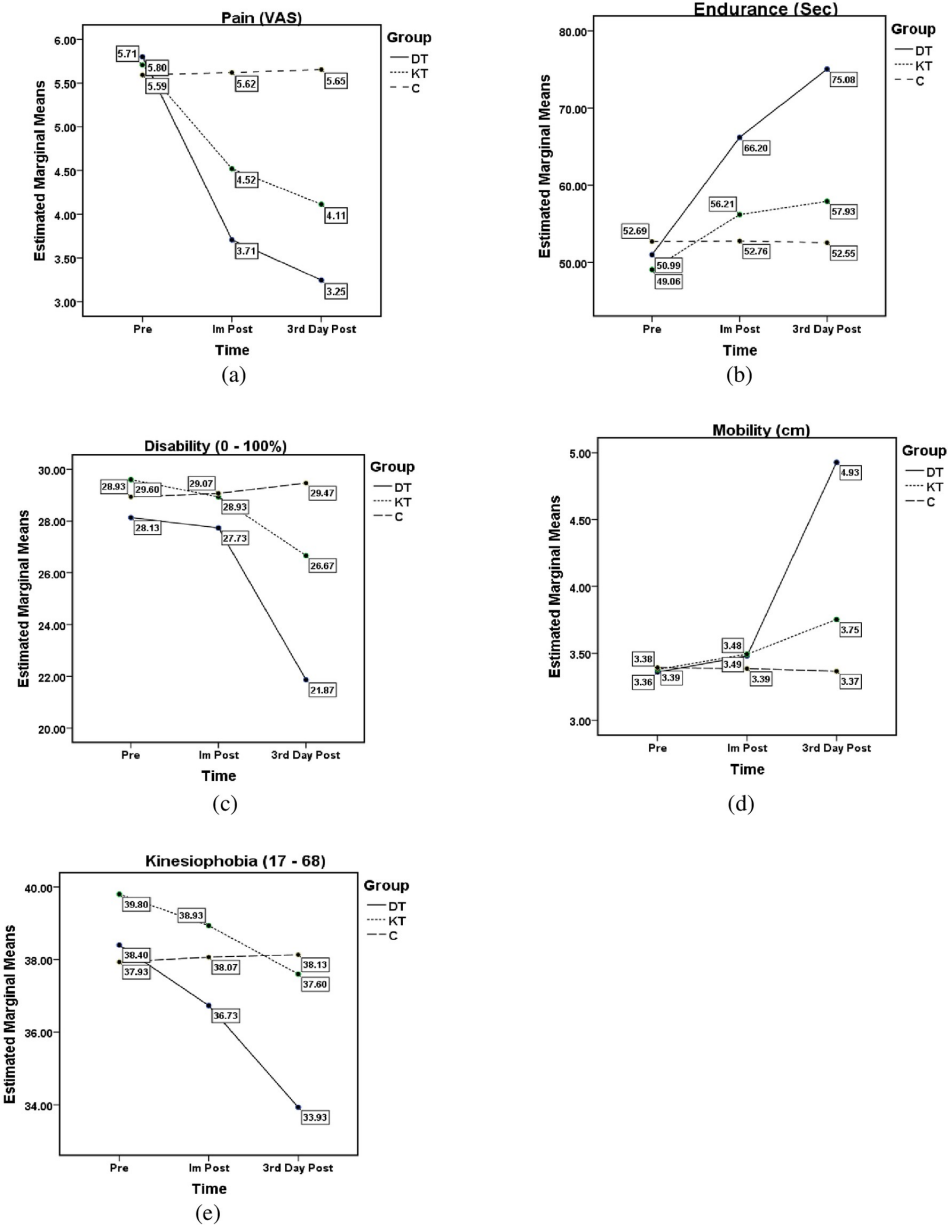
Group and time interaction of outcome variables. (a). Pain; (b). Endurance; (c). Disability; (d). Mobility; and (e). Kinesiophobia. VAS = visual analog scale, DT = Dynamic type group, KT = Kinesio tape group, C = control group, Im Post = immediately post.

Mixed methods ANOVAs showed a significant difference between groups and time for pain, endurance, mobility, and kinesiophobia, whereas the disability showed a significant difference only in time intervals (Table 3). The time and the group interaction effect are shown in Fig 3 for all the variables.

**Table 3:** Summary of statistical results of the mixed ANOVA and mean ± SD of all outcome variables.

Variables	Groups	Mean ± SD			Mixed ANOVA (*p*-value)		
		Pre	Immediate post	3^rd^ Day post	Interaction (Group and Time) effect η_p_^2^ (*p*-value)	Group effect η_p_^2^ (*p*-value)	Time effect η_p_^2^ (*p*-value)
Pain (VAS 0 to 10)	DT	5.80 ± 0.87	3.71 ± 0.82	3.25 ± 0.93	0.550 (<0.001)*	0.398 (<0.001)*	0.659 (<0.001)*
	KT	5.71 ± 0.88	4.52 ± 0.93	4.11 ± 0.92			
	C	5.59 ± 0.72	5.62 ± 0.72	5.65 ± 0.73			
Endurance (sec)	DT	50.99 ± 11.07	66.19 ± 10.29	75.08 ± 11.54	0.768 (<0.001)*	0.232 (0.004)*	0.802 (<0.001)*
	KT	49.06 ± 10.34	56.21 ± 8.93	57.93 ± 9.58			
	C	52.69 ± 8.32	52.76 ± 8.32	52.55 ± 8.88			
Disability (0–100%)	DT	28.13 ± 4.24	27.73 ± 4.27	21.87 ± 4.10	0.638 (<0.001)*	0.096 (0.120)	0.648 (<0.001)*
	KT	29.60 ± 5.19	28.93 ± 5.59	26.67 ± 4.45			
	C	28.93 ± 4.19	29.07 ± 4.27	29.47 ± 4.03			
Mobility (cm)	DT	3.36 ± 0.45	3.48 ± 0.48	4.93 ± 0.55	0.711 (<0.001)*	0.218 (0.006)*	0.675 (<0.001)*
	KT	3.38 ± 0.45	3.49 ± 0.46	3.75 ± 0.67			
	C	3.39 ± 0.44	3.39 ± 0.42	3.37 ± 0.43			
Kinesiophobia (17–68)	DT	38.40 ± 2.06	36.73 ± 1.94	33.93 ± 2.60	0.562 (<0.001)*	0.152 (0.031)*	0.623 (<0.001)*
	KT	39.80 ± 3.61	38.93 ± 3.53	37.60 ± 3.31			
	C	37.93 ± 1.75	38.07 ± 1.94	38.13 ± 1.36			

ANOVA = analysis of variance, VAS = visual analog scale, DT = dynamic tape, KT = kinesio tape, C = control.

* Significant difference: p <0.05.

As stated, we used mixed methods ANOVAs and employed the Tukey post hoc criterion for significance. Our findings indicated that both the DT (*p*<0.001) and KT (*p* = 0.008) significantly decreased pain than no tape. The DT significantly helped increase back extensor endurance than KT (*p* = 0.023) and no tape (*p* = 0.006). There is no significant difference noticed in disability among all three groups (p>0.05). The DT revealed a significant improvement in spinal mobility than no tape (*p* = 0.005) and no significant improvement seen with KT (p = 0.072). Both DT and KT did not significantly improve than no tape (p>0.05) in kinesiophobia. No harmful or adverse effects were observed in any of the groups throughout the study and every patient in all three groups completed the study.

## Discussion

This randomized controlled trial aimed to compare the effectiveness of DT to KT and controls in patients with CNLBP using the outcomes of pain, endurance, disability, mobility, and kinesiophobia. To our knowledge, this is the first study to analyze the effect of DT on CNLBP. This study’s hypothesis that the improvement would be found in the DT group compared to the KT group and the control group in the outcome variables, was not confirmed except with the endurance of back extensor muscle.

We intentionally not included sham taping applications with control group. Studies have well documented the strong effect of sham or placebo taping application on outcome variables [47, 48]. The sham taping use may produce a placebo effect on subjective outcome measures, particularly with pain, disability, and kinesiophobia [49].

Taping is commonly used as an assistive technique in preventing and managing musculoskeletal conditions [50]. Among all the available types of tape, KT is widely used to manage clinical conditions and clinical research. An examination of the available systematic reviews [25, 51–53] to evaluate KT’s effectiveness on CNLBP reveals that there is no current evidence to support the use of this method. At the same time, we need to examine another tape, such as DT, that manages CNLBP.

The low back muscle’s eccentric contraction is extremely complicated while lifting objects or from forward bending to erect posture in subjects with LBP [54]. Back extensor muscles often have to generate an amazingly large force to lift the body and/or hold heavy objects [55]. It is highly important to control the maximum torque generated by the spinal extensor musculature during the eccentric and concentric contraction in day-to-day activities and to minimize the effect of external load or bodyweight on back extensors for patients with CNLBP [54]. The DT has the quality of a mechanical effect to decelerate the eccentric work, assisting the concentric movement, and absorbing the load placed on muscles during simple and complex movements [19]. Thus, we aimed to assess the immediate and short term effectiveness of DT on pain, endurance, disability, mobility, and kinesiophobia among patients with CNLBP. The results showed significant (p<0.05) immediate and short-term improvement only in back extensor endurance among individuals with CNLBP in favor of DT compared to KT or no tape. Among the other outcome comparisons, the DT group did not show any statistical significance, indicating improvements only in the group who underwent DT.

Application of therapeutic tape on the skin produces sufficient proprioceptive stimulus to generate cutaneous mechanoreceptor inputs to the central nervous system, which may decrease the nociceptive inputs and activate the descending pain inhibitory system [56]. However, the proposed neurophysiologic mechanism of tape application is the reason for the decrease in pain immediately after the application of tape. Thus, both the tapes significantly decreased pain than no tape. Moreover, studies using DT alone or with therapeutic intervention have shown a meaningful reduction of pain among subjects with GTPS [32], post-operative shoulder [33], mechanical neck pain [34], and knee osteoarthritis [57] which corroborates the present study. Not only, but also studies using KT to decrease pain among subjects with chronic LBP in short to medium duration produced a significant reduction of pain [21, 26].

The association between paravertebral muscle fatigue and LBP has been extensively studied. Delayed muscle activation, decreased ratio between lumbar flexors and extensors, or resistance to fatigue of the lumbar extensor muscles are the factors responsible for developing chronic LBP [58]. Therefore, it is essential to measure the endurance or resistance to the trunk extensor’s fatigue, as developing appropriate management is of high clinical importance for patients with CNLBP. In this study, the only outcome of endurance showed significant improvement with DT application than KT application and no tape on immediate and short-term assessment among individuals with CNLBP.

The DT is applied when the joint is in a shortened position; once the joint or muscle lengthens, the tape stretches further, and the absorbed energy is stored as elastic potential energy. During day-to-day activities, the muscle shortens, and the stored energy is then transferred back into kinetic energy. As a result, this assists the muscle’s movement, while the energy transfer leads to decreased workload, improved biomechanical efficiency, and improved fatigue tolerance by the working muscles [59]. Besides, DT controls and absorbs the eccentric load on back extensors, which is why the beneficial effect is immediate and short-term. Moreover, KT’s post-application on non-specific LBP showed no improvement than rigid therapeutic tape on back muscle endurance [39] which also supports the present study. However, the immediate post KT application produced better back muscle endurance than the placebo tape or no tape only among the healthy subjects [60].

In terms of disability, it is a crucial issue in LBP, affecting physical performance and work productivity. Clinicians must address the importance of minimizing disability-related problems during their rehabilitation in CNLBP. About disability outcome, the present study demonstrates no immediate and short-term effect of both DT and KT application on CNLBP. Moreover, researchers have evaluated the impact of DT application with other therapeutic interventions on disability among patients with mechanical neck pain and post-operative shoulder over three weeks and found a significant reduction in disability scores [33, 34]. The immediate effects of DT application on disability-related outcomes have mixed results. Bittencourt et al. [35] found significant immediate post-application improvement in female volleyball athletes’ frontal plane knee projection angle.

In contrast, Bek et al. [57] found no significant immediate post-application improvement of DT on functional performance among subjects with knee osteoarthritis. Studies also analyzed the short to median-term effects of KT on disability among individuals with CNLBP and produced mixed results. Studies conducted by Macedo et al. [26], Castro-Sanchez et al. [21], Al-Shareef et al. [61], and Koroglu et al. [46] concluded that KT helps to improve disability. In contrast, studies carried out by Luz et al. [28] and Kamali et al. [62] concluded that KT was not helped to improve disability.

This study’s secondary outcomes are the mobility of the spine (ROM) and kinesiophobia (movement fear avoidance). LBP patients often have reduced ROM/mobility in the lumbar spine [63]. It has been suggested that LBP leads to limited mobility of the spine through the unwillingness of the injured individual to move their trunk to the end range due to the fear of increasing pain [64]. Until now, only two studies have evaluated the effects of DT application on ROM among subjects with postoperative shoulder and chronic mechanical neck pain, both find significant improvement in ROM over three weeks [33, 34]. Studies also examined the short to medium-term effects of KT application on ROM among individuals with CNLBP. They concluded as KT is favorable [21, 46, 61] and unfavorable [26, 63] for the improvement of ROM. The current study also assessed the effect of tapes on the lumbar flexion range and found a significant improvement with both tapes on immediate and short-term measurements. The possible reason to increase ROM after applying tapes would be the stimulation of large diameter afferent fibers, which modulates nociceptor input to inhibit pain transmission [23] and the neural feedback, which may inhibit the mechanical irritation of soft tissues when moving the lumbar spine [21].

Pain-related fear of movement/kinesiophobia is significantly associated with restricted physical performance among patients with chronic LBP [65]. Clinicians should also focus on the alleviation of pain-related fear of movement to get better outcomes in rehabilitation. A study done by Castro-Sanchez et al. was investigated the effect of KT on CNLBP and found no significant improvement between the KT group and the sham taping group in medium to long term duration. Like this, the present study also shows that both DT and KT produce no significant reduction in kinesiophobia than control groups.

The present study also reports several limitations. First, we assessed the immediate and short-term effects of the DT application. Therefore, the effects of long-term or repeated use of DT remain unknown. Second, only male patients participated in this study due to the country’s cultural norms where it took place. Third, the amount of stretch in the KT was determined at 10–15%, and the same was achieved or not known by the therapist during the tape application. Fourth, the sham taping was not included in this trial which may help compare the therapeutic effects of both DT and KT with sham taping. Future research should assess DT’s effects on long-term outcomes for individuals with CNLBP and should address these limitations.

## Conclusion

This randomized controlled trial showed that the DT does not have a significant additional effect on pain, disability, mobility, and kinesiophobia among individuals with CNLBP compared to KT. However, the participants experienced significant improvement in back muscular endurance after the application of DT. This finding suggests that DT controls the processes that lead to back muscle fatigue. Therefore, more studies are required to examine the therapeutic benefits of DT in treating patients with CNLBP.

## Supporting information

S1 Protocol (DOCX)

S1 Checklist CONSORT 2010 checklist of information to include when reporting a randomised trial*. (PDF)

S2 Checklist (PDF)
