# Supplementary material for: The immediate and short-term effects of dynamic taping on pain, endurance, disability, mobility and kinesiophobia in individuals with chronic non-specific low back pain: A randomized controlled trial
Source: PLoS One. 2020 Sep 29;15(9):e0239505. doi: 10.1371/journal.pone.0239505 (PMC7523973; doi:10.1371/journal.pone.0239505)
Supplement: S1 Protocol — (DOCX) [file pone.0239505.s001.docx]

**Immediate and short-term effects of Dynamic taping on pain, disability, mobility and endurance among subjects with chronic non-specific low back pain – A randomised controlled trail**

**Introduction:**

Low back pain (LBP) is a significant public health condition and it is associated with a high rate of absenteeism from work, disability, and frequent use of health services. Current literature provides several possibilities for the treatment of LBP that vary according to duration of symptoms and classification of this condition. These treatments range from educational programs to behavioral cognitive therapy, medication, electro physical agents, manual therapy, general exercises and specific spinal stabilization exercises, among others.

Although the aforementioned treatments have been extensively adopted, they exert a moderate effect at most, with recurrences typically noted. Therefore, patients and clinicians critically require more effective therapeutic approaches.

Several types of tape and their associated application methods are available, with different underlying philosophies regarding their modes of action. Kinesio tape, Dynamic tape, Rigid tape, Micropore tape, Athletic tape and many other tapes are available to manage and/or rehabilitate the injuries.

Dynamic taping (DT) is a relatively new treatment tech­nique, which is increasingly becoming an adjunctive method to treat musculoskeletal problems. In the year 2009, Kendrick produced DT which is made up of visco-elastic nylon and lycra blend material which has ability to stretch in four directions, strong elastic resistance and recoil, high degree of stretch (more than 200%) with no rigid end point and visco-elastic properties. The primary mode of action of DT is mechanical (deceleration of eccentric action, load absorption and assistance of movement) and the secondary mode of action is neurophysiological.

However, no research has evaluated the effects of DT on LBP and the aim of the present study is to determine the effect of DT in the treatment of chronic non-specific low back pain (chronic NSLBP).

**Aims & Objectives:**

1. The main objective of this study is to assess the immediate effectiveness of DT application on the reduction of pain, functional disability, range of motion (ROM) and endurance in participants with chronic non specific low back pain.
2. The secondary aim of this study is to assess the short-term (end of 3^rd^ day) effectiveness of DT application on the reduction of pain, functional disability, range of motion (ROM) and endurance in participants with chronic non specific low back pain.

### Study Population:

**Inclusion criteria**:

- Patients with LBP of at least 30 days in duration and aged between 18 and 60 years were included.
- Pain intensity with minimum scoring of 3 on a Visual Analog Scale (VAS)
- Disability with minimum scoring of 20 out of 100 in Oswestry Disability Index (ODI)

**Exclusion criteria:**

- Patients with any contraindications to the use of taping (skin diseases)
- Pain radiating to the knee
- Known or suspected serious congenital or acquired spinal pathology, spinal surgery hisoty, lumbar disc herniation, rheumatoid arthritis or spondyloarthropathy diagnosis.
- Unable to tolerate Biering-Sorensen test.

**Methodology:**

Sample size: Forty five subjects with chronic NSLBP.

Study design: Randomized controlled trail

Target number of participants: 45

Procedure

Participants are requested to join this study while they are at an out-patient physiotherapy clinic. Initial examination/assessment is done for inclusion in the study. A small piece of Dynamic tape (DT) and Kinesiology tape (KT) is applied to the right and left forearms, respectively. The next day (after 24 hours), the researcher examines the forearm and makes sure that there is no allergic reaction. If any allergic reactions present then the participant is excluded from the study. The pre-test assessment is carried out and the participants are randomly allocated to one of three groups. Dynamic tape is applied parallel to the spine from the posterior superior iliac spine (PSIS) to T12 thoracic vertebra for the first group (Experimental Group 1). For those in the second group (Experimental Group 2), the Kinesiology tape is applied from the posterior superior iliac spine (PSIS) to T12 thoracic vertebra. No treatment is received by the control group. After the application of the tapes, the patients undergo the same pre-test measurements after 2 hours of tape application. The participants are instructed to re-visit after 3 days and the measurements are taken in the presence of tape. After completing the measurements, the routine treatment is given to all the participants (experimental group 1, experimental group 2, and control group).

Triage to determine

participant

eligibility

**Day-1**

Initial assessment;

Informed consent.

**Total Duration – 5 days**

**Day-3**

**Day-2**

**After 3 days**

Randomisation

Control group

Sham taping group

Dynamic taping group

Allergy test (24 hour application)

Base line assessment

1. Pain
2. Disability
3. Endurance
4. Mobility
5. Kinesiophobia

Immediate post taping assessment

1. Pain
2. Disability
3. Endurance
4. Mobility
5. Kinesiophobia

Follow-up assessment

1. Pain
2. Disability
3. Endurance
4. Mobility
5. Kinesiophobia

Outcome Measures:

1. **Pain:** Pain intensity is recorded by the participant using a 10-cm visual analogue scale (VAS), where 0 represented no pain and 10 represented unbearable pain.
2. **Disability**: The English / Arabic version of Oswestry Disability Index (ODI) will be used. It is a self-rating questionnaire used to evaluate functional physical disability. It includes 10 sections of six propositions and each rated on a 0 – 5 scale. Relative values are reported (total score / total possible score X 100%). Higher score indicate worse disability.
3. **Endurance:** Back extensor muscle endurance assessed using by Biering-Sorensen test. It has been established good test-retest reliability (intraclass correlation coefficient = 0.88; standard error of measurement, 11.6 seconds in patients with LBP), validity, and responsiveness. Participants are positioned on a treatment table in prone, with the lower half of their body secured with 3 straps.

For testing, the participant’s ability to maintain a horizontal position is timed using a stopwatch/timer, and standardized verbal encouragement is provided at 30-second intervals.

The participant is placed in the starting position for the test, prone on a plinth with the upper edge of the iliac crests aligned with the edge of the table. A second hydraulic table is transversely placed at the same height to the first one under the trunk and upper body, in order that the participants may be supported completely in prone position before initiation of the test. The lower limbs are fixed to the table in full extension, together, and with ankles in plantar flexion using three straps perpendicular to the midline.

The first strap is located at the level of the greater trochanter, the second one at the level of the popliteal fossa and the last one at the level of the Achilles tendon insertion as close as possible to the malleoli. An inclinometer fixed to the participant’s inter-scapular region by an elastic strap around the chest, is used to measure changes in flexion or extension of the subjects during the test.

On initiation of the test the second table is lowered and the subject is requested to place their arms across their chest and maintain a neutral spinal position The timer is started as soon as arms are positioned across the chest and the participant is maintaining this position without assistance However at no point is either the researcher or participant aware of the amount of time that had passed, as this has been shown to be a factor directly influencing test results.

An oscillation of the inclinometer needle of 10^0^ during the test is permitted, between 5^0^ of extension and 5^0^ of flexion. The test ended when any part of the participant’s upper limb touched the table or when they are unable to recover the test position even with verbal encouragement . At this point the timer is stopped and the test finished. The duration of the test documented in seconds.

1. **Mobility**: Lumbar / back flexion, extension, rotations are measured using… back range of motion device (BROM).
   1. Back range of motion (BROM) device
   2. Inclinometer
   3. Finger to ground distance
   4. Modified Schober test
2. **Movement fear avoidance**: The English/Arabic version of the Tampa Scale of Kinesiophobia (TSK) is a 17-item self report checklist using a 4-point Likert scale that was developed as a measure of fear of movement or (re)injury. Kinesiophobia is defined by the developers as “an irrational and debilitating fear of physical movement and activity resulting from a feeling of vulnerability to painful injury or re-injury. The total score ranges between 17 and 68. A high value on the TSK indicates a high degree of kinesiophobia 37 or over is considered a high score, while scores below 37 are considered low.
