# Supplementary material for: The immediate and short-term effects of dynamic taping on pain, endurance, disability, mobility and kinesiophobia in individuals with chronic non-specific low back pain: A randomized controlled trial
Source: PLoS One. 2020 Sep 29;15(9):e0239505. doi: 10.1371/journal.pone.0239505 (PMC7523973; doi:10.1371/journal.pone.0239505)
Supplement: S1 Checklist — (PDF) [file pone.0239505.s002.pdf]

CONSORT 2010 checklist of information to include when reporting a randomised trial*

Section/Topic	Item No	Checklist item	Reported on page No
**Title and abstract**			
	1a	Identification as a randomised trial in the title	1
	1b	Structured summary of trial design, methods, results, and conclusions (for specific guidance see CONSORT for abstracts)	2
**Introduction**			
Background and objectives	2a	Scientific background and explanation of rationale	3, 4 and 5
	2b	Specific objectives or hypotheses	5
**Methods**			
Trial design	3a	Description of trial design (such as parallel, factorial) including allocation ratio	5, 6 and 7.
	3b	Important changes to methods after trial commencement (such as eligibility criteria), with reasons	7
Participants	4a	Eligibility criteria for participants	5 and 6
	4b	Settings and locations where the data were collected	5
Interventions	5	The interventions for each group with sufficient details to allow replication, including how and when they were actually administered	10 and 11
Outcomes	6a	Completely defined pre-specified primary and secondary outcome measures, including how and when they were assessed	7, 8, 9 and 10
	6b	Any changes to trial outcomes after the trial commenced, with reasons	7
Sample size	7a	How sample size was determined	11
	7b	When applicable, explanation of any interim analyses and stopping guidelines	11
Randomisation:			
Sequence generation	8a	Method used to generate the random allocation sequence	6 and 7
	8b	Type of randomisation; details of any restriction (such as blocking and block size)	6
Allocation concealment mechanism	9	Mechanism used to implement the random allocation sequence (such as sequentially numbered containers), describing any steps taken to conceal the sequence until interventions were assigned	6 and 7
Implementation	10	Who generated the random allocation sequence, who enrolled participants, and who assigned participants to interventions	6 and 7
Blinding	11a	If done, who was blinded after assignment to interventions (for example, participants, care providers, those	6 and 7
