# Supplementary material for: The immediate and short-term effects of dynamic taping on pain, endurance, disability, mobility and kinesiophobia in individuals with chronic non-specific low back pain: A randomized controlled trial
Source: PLoS One. 2020 Sep 29;15(9):e0239505. doi: 10.1371/journal.pone.0239505 (PMC7523973; doi:10.1371/journal.pone.0239505)
Supplement: S2 Checklist — (PDF) [file pone.0239505.s003.pdf]

Section/Topic	Item No	Checklist item	Reported on page No
		assessing outcomes) and how	
	11b	If relevant, description of the similarity of interventions	10 and 11
Statistical methods	12a	Statistical methods used to compare groups for primary and secondary outcomes	11 and 12
	12b	Methods for additional analyses, such as subgroup analyses and adjusted analyses	12
**Results**			
Participant flow (a diagram is strongly recommended)	13a	For each group, the numbers of participants who were randomly assigned, received intended treatment, and were analysed for the primary outcome	12 and 13
	13b	For each group, losses and exclusions after randomisation, together with reasons	12
Recruitment	14a	Dates defining the periods of recruitment and follow-up	12
	14b	Why the trial ended or was stopped	-
Baseline data	15	A table showing baseline demographic and clinical characteristics for each group	13
Numbers analysed	16	For each group, number of participants (denominator) included in each analysis and whether the analysis was by original assigned groups	12 and 13
Outcomes and estimation	17a	For each primary and secondary outcome, results for each group, and the estimated effect size and its precision (such as 95% confidence interval)	14 and 15
	17b	For binary outcomes, presentation of both absolute and relative effect sizes is recommended	14
Ancillary analyses	18	Results of any other analyses performed, including subgroup analyses and adjusted analyses, distinguishing pre-specified from exploratory	15 and 16
Harms	19	All important harms or unintended effects in each group (for specific guidance see CONSORT for harms)	16
**Discussion**			
Limitations	20	Trial limitations, addressing sources of potential bias, imprecision, and, if relevant, multiplicity of analyses	20 and 21
Generalisability	21	Generalisability (external validity, applicability) of the trial findings	21
Interpretation	22	Interpretation consistent with results, balancing benefits and harms, and considering other relevant evidence	16, 17, 18, 19 and 20
**Other information**			
Registration	23	Registration number and name of trial registry	6
Protocol	24	Where the full trial protocol can be accessed, if available	-
Funding	25	Sources of funding and other support (such as supply of drugs), role of funders	Included

*We strongly recommend reading this statement in conjunction with the CONSORT 2010 Explanation and Elaboration for important clarifications on all the items. If relevant, we also recommend reading CONSORT extensions for cluster randomised trials, non-inferiority and equivalence trials, non-pharmacological treatments, herbal interventions, and pragmatic trials. Additional extensions are forthcoming: for those and for up to date references relevant to this checklist, see www.consort-statement.org.
